# Genome-wide chromatin accessibility analyses provide a map for enhancing optic nerve regeneration

**DOI:** 10.1038/s41598-021-94341-y

**Published:** 2021-07-21

**Authors:** Wolfgang Pita-Thomas, Tassia Mangetti Gonçalves, Ajeet Kumar, Guoyan Zhao, Valeria Cavalli

**Affiliations:** 1 Department of Neuroscience, Washington University School of Medicine, St Louis, MO 63110 USA; 2 Department of Pathology and Immunology, Washington University School of Medicine, St Louis, MO 63110 USA; 3 Hope Center for Neurological Disorders, Washington University School of Medicine, St. Louis, MO 63110 USA; 4 Center of Regenerative Medicine, Washington University School of Medicine, St. Louis, MO 63110 USA

**Keywords:** Epigenetics in the nervous system, Regeneration and repair in the nervous system

## Abstract

Retinal Ganglion Cells (RGCs) lose their ability to grow axons during development. Adult RGCs thus fail to regenerate their axons after injury, leading to vision loss. To uncover mechanisms that promote regeneration of RGC axons, we identified transcription factors (TF) and open chromatin regions that are enriched in rat embryonic RGCs (high axon growth capacity) compared to postnatal RGCs (low axon growth capacity). We found that developmental stage-specific gene expression changes correlated with changes in promoter chromatin accessibility. Binding motifs for TFs such as CREB, CTCF, JUN and YY1 were enriched in the regions of the chromatin that were more accessible in embryonic RGCs. Proteomic analysis of purified rat RGC nuclei confirmed the expression of TFs with potential role in axon growth such as CREB, CTCF, YY1, and JUND. The CREB/ATF binding motif was widespread at the open chromatin region of known pro-regenerative TFs, supporting a role of CREB in regulating axon regeneration. Consistently, overexpression of CREB fused to the VP64 transactivation domain in mouse RGCs promoted axon regeneration after optic nerve injury. Our study provides a map of the chromatin accessibility during RGC development and highlights that TF associated with developmental axon growth can stimulate axon regeneration in mature RGC.

## Introduction

Retinal Ganglion Cells (RGCs) in the retina receive visual information from bipolar cells and transmit it to several regions in the brain via axons projecting into the optic nerve. RGCs originate from retinal precursor cells and most of them are born between E11 and E20^1^. Transcription factors (TFs) such as Pax6, Math5, Pou4f2, form a hierarchical network that regulates RGC differentiation from retinal precursors in space and time. Once differentiated, RGCs grow their axons towards the brain through the optic nerve. Early born RGCs (born before E16) arrive at the superior colliculus before birth while late born RGCs (born after E16) arrive at the superior colliculus at P4/P5^2^. Right before eye-opening (P12), all RGCs have formed stable synapses with their brain targets, providing a functional visual system. The maturation of the visual system correlates with a decrease in the regenerative capacity of RGCs. Therefore, RGC injury in adult mammals is followed by axon regeneration failure and a degeneration process that leads to cell death. As a result, diseases that cause optic nerve damage such as traumatic optic neuropathy, glaucoma, or optic nerve ischemia result in an irreversible loss of vision. Identifying molecular and cellular mechanisms that increase survival and regeneration of RGC may offer new treatment strategies for patients with glaucoma or other types of optic neuropathies.

RGCs lose their capacity to grow axons after birth^3,4^. Axon growth capacity in rat RGCs peak at embryonic day 21 (E21), and start declining after birth reaching their minimum growth potential at postnatal day 11 (P11), right before eye-opening^4^ suggesting that the axon growth program is substituted by a synapse function program. Isolation of RGCs by immunopanning^5^, laser microdissection^6^, and cell sorting^7^ has enabled the identification of gene expression changes between these developmental stages and the differences in axon growth capacity between RGC subtypes. Multiple TFs that promote axon growth, such as CREB^6^, SOX11^8^ and KLF7^5^ are downregulated during RGCs development. Other TFs that inhibit axon growth such as KLF4 and KLF9 are upregulated during development^5^. Overexpressing KLF7 and Sox11, or deleting KLF4 and KLF9 promotes axon regeneration after optic nerve injury^5,8–11^. Activating the CREB pathway by injecting compounds that mimic cAMP promotes modest regeneration after optic nerve injury^12,13^. Overexpressing CREB fused to the VP16 transactivation domain promotes some axon regeneration in sensory neurons after spinal cord injury^14^, but the effect of CREB expression in RGCs has not been tested. It has been shown that chromatin accessibility of some TF binding sites, such as KLF7 binding site, is limited in mature cortical neurons, and that the transactivation domain VP16 is needed to boost KLF7 activity in promoting regeneration^9,15^. Therefore, chromatin accessibility may represent another epigenetic mechanism controlling the axon regeneration program. Proteins that modify chromatin accessibility, such as histone deacetylases and histone acetyltransferase have also been shown to play a role in sensory neuron^16–20^ and RGC^21–24^ regeneration. The interplay of TFs and chromatin accessibility regulates the expression levels of downstream genes such as *Gap43* and *Tubb3* whose final location is at the growth cone^25^. Some of these downstream genes may also regulate signaling pathways that control metabolism. Activating pathways that control endosome recycling, cell growth, and ribosome biogenesis such as overexpressing protrudin-1^26^ to mobilize endosomes into axons or deleting key regulators such as PTEN, IL22 or SOCS3 to stimulate the mTOR and STAT3 pathways^27–29^ promotes abundant axon regeneration. Therefore, the interactions of genes that control growth cone dynamics and cell metabolism increase the axon growth capacity of RGCs. Altogether, these studies point to the complex network of regenerative associated genes, epigenetics, TFs and downstream genes regulating the decline in axon growth capacity during RGC development.

Analysis of chromatin accessibility has previously resulted in the prediction of target genes of TFs, the hierarchy of TFs in a given network, and the discovery of TFs with novel roles in developmental biology^30^. Understanding the hierarchical order of these TFs and unraveling the chromatin accessibility at different developmental stages may allow the reprogramming of adult RGCs for axon growth. Here we performed a comprehensive characterization of the gene expression, chromatin accessibility and nuclear proteome during the critical developmental period of axon growth decline in rat RGCs (E21 vs P11) using RNA-seq, ATAC-seq (Assay for Transposase-Accessible Chromatin using sequencing) and liquid chromatography–mass spectrometry (LC–MS). We found that gene expression changes correlate with chromatin accessibility changes at the promoter region. Proteomic analysis identified the expression of TFs with potential role in axon growth such as CREB, CTCF, YY1, and JUND. Interestingly, CREB binding sites were significantly enriched in the open chromatin regions specific to the E21 RGCs. Overexpressing CREB fused to a VP64 transactivation domain in RGCs induced axon regeneration after optic nerve injury, mimicking the axon growth capacity of embryonic neurons. Our data provides a road map of the chromatin accessibility during RGC development and highlights that manipulating TFs associated with developmental stages with high growth potential can stimulate axon growth in adulthood.

## Results

### Genetic programs that control axon growth are downregulated during RGC development

**Figure 1:**
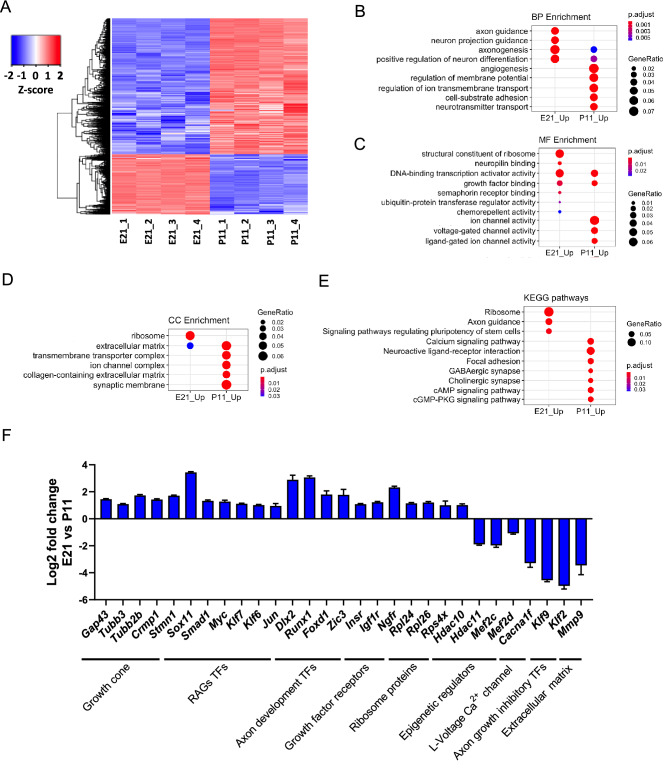
RNA-seq analysis of E21 and P11 purified RGCs. **(A)** Differentially expressed genes (DEGs) between E21 and P11. The cut off for DEGs displayed in the figure was log2 fold change > 1 and false discovery rate (FDR) adjusted p values < 0.01, which includes a Benjamini–Hochberg correction. Red represents gene expression above average expression level across all samples. Blue represents gene expression below average expression level across all samples. A total of 3,646 genes were differentially expressed, 1116 upregulated and 2530 downregulated in E21. **(B–D)** Gene ontology (GO) analysis of the DEGs. A subset of the most  significantly enriched GO terms in **(B)** biological processes (BP), **(C)** molecular functions (MF) and **(D)** cellular component (CC) are represented. GO terms with a FDR corrected p value < 0.05 were considered as significant. **(E)** KEGG pathways analysis of DEGs. A subset of the twelve most significantly enriched pathways are represented. **(F)** Gene expression change between E21 and P11 of key individual genes from the most relevant pathways to axon growth in **(B–D)**.

To determine the transcriptional changes that occur between embryonic 21 days (E21) and postnatal 11 days (P11) RGCs, RNA was extracted from immunopanned RGCs and sequenced in four independent replicates. We identified 3,646 genes differentially regulated (log_2_ fold change > 1 and Benjamini–Hochberg corrected p value < 0.01) between E21 and P11 RGCs (Fig. 1A, Supplementary Fig. 1A, and Supplementary File 1). There were 1,116 genes differentially upregulated and 2,530 genes downregulated at E21 (Supplementary Fig. 1B). RNA expression analysis showed that RGC marker genes such as *Pou4f1* and *RBPMS* were highly enriched compared to markers of microglia, astrocyte, Muller cells, photoreceptor, bipolar, and amacrine cells (Supplementary Fig. 1C), as expected from previous publications reporting 99% purity of RGCs derived from this immunopanning technique^4^. We also observed that many of the marker genes for RGC subtypes identified by single cell sequencing, such as *Opn4* (intrinsically photosensitive RGCs, ipRGCs), *Nefh* (αRGCs) and *Cartpt* (ON–OFF directional sensitive RGCs, ooDSGCs)^7^, were expressed at lower levels at E21 and were significantly upregulated at P11 (Supplementary Fig. 1C ). This suggests that E21 RGCs are not fully differentiated and that RGCs lose their capacity to grow axons during their differentiation into different subtypes. Interestingly, some RGC subtypes such as ipRGCs and, especially, αRGCs retain some axon growth capacity while others such as ooDSGCs do not^7,31^.

Gene Ontology (GO) analysis revealed that Biological Processes (BP) related to axon guidance and axonogenesis are enriched at E21 (Fig. 1B, Supplementary File 2), including genes whose protein products are localized in RGC growth cones such as CRMP1, STMN1, GAP43, and β-tubulins, aligning with an essential role in axon growth and pathfinding in E21 RGCs (Fig. 1F). GO Molecular Function revealed enrichment at P11 of genes related to voltage-gated channels, including the L-type calcium channel Cav 1.4 subunit (*Cacna1f*) (Fig. 1C,F, Supplementary File 2) aligning with their role in synapse function and neuronal activity at P11. It is noteworthy that prior studies have shown that voltage gated ion channels, specifically L-type calcium channels, suppress axon regeneration in sensory neurons^32^. GO analysis for Molecular Function also showed an enrichment of genes related to transcription factors at both E21 and P11 (Fig. 1C, Supplementary File 2). We observed E21 upregulation of TF genes with known role in optic nerve axon regeneration such as *Sox11*, *Klf6*, *Myc*, and *Klf7*, and also other TFs such as *Dlx2*, *Runx1*, *Foxd1*, *Zic3*, and *Isl1* with described role in axon growth development. Interestingly, αRGCs have higher levels of *Myc* in postnatal stages compared to other subtypes^33^, suggesting high Myc expression levels in αRGCs may partially explain their higher axon growth capacity. We did not observe major changes in genes related to epigenetic regulation, with few exceptions such as *HDAC10,* which is upregulated at E21 and *HDAC11,* which *is* downregulated at E21. We also observed postnatal upregulation of the MEF2 family of TFs, which are known to work together with class IIa HDACs such as HDAC5^34^. Whereas HDAC5 promotes optic nerve regeneration^23^, deleting MEF2 members promotes RGC survival but not axon regeneration after optic nerve injury^35^. The Molecular Function GO analysis also showed enrichment of genes related to growth factor binding. We observed some key growth factor receptors genes upregulated in E21 such as *Insr*, *Igf1r* and *Ngfr*, which may contribute to a higher activation of PI3K/mTOR pathway at this stage providing a higher axon growth potential. Surprisingly, we did not observe a differential expression of genes related to lipid metabolism such as protrudin-1 (*Zfyve27*) suggesting that these changes may happen later during development. Cellular components (CC) enriched at E21 are predominantly related to ribosomes suggesting active cell growth at this stage (Fig. 1D, Supplementary File 2). A decline in ribosome gene expression has been observed during neuronal development and has been related to the loss of axon growth capacity^36^. Multiple ribosome genes are downregulated in P11 including *Rpl24* and *Rpl26*, which have been previously implicated in axon growth decline during development^37^. Genes related to extracellular matrix were also enriched at P11 (Fig. 1D, Supplementary File 2) suggesting that RGCs regulate their surrounding extracellular matrix during development. We observed that P11 RGCs upregulate matrix metalloproteinase 9 (*MMP9*) which is known to regulate cell death in these cells^38^. Whether the interplay between RGCs and surrounding cells during development affects axon growth capacity is unknown. KEGG (Kyoto Encyclopedia of Genes and Genomes biological) pathway analysis^39–41^ corroborated that pathways related to ribosome function and axon guidance are enriched in E21, whereas neurophysiological pathways typical of synapse function are enriched in P11 (Fig. 1E, Supplementary File 2). This transcriptional analysis reveals that, as expected, E21 RGCs are in an active axon growth stage, whereas P11 RGCs have established synapses and downregulated the axon growth program.

### Changes in gene expression correlate with changes in chromatin accessibility at the promoter regions during RGC development

**Figure 2:**
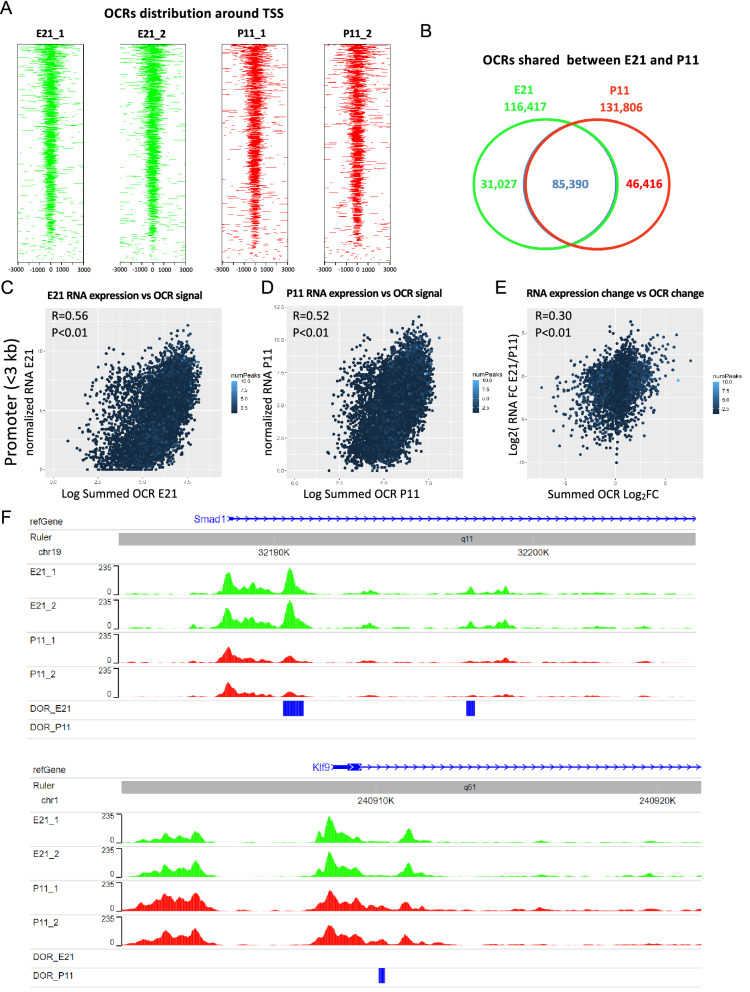
Chromatin accessibility changes at the promoter region, defined as < 3 kb from the transcription start site (TSS), correlate with gene expression changes. **(A)** The distribution of open chromatin regions (OCRs) with respect to the closest gene TSS in the 4 RGC samples is represented. The number of genes plotted for each sample was 11,310 (E21_1), 11,572 (E21_2), 11,449 (P11_1), and 11,542 (P11_2). Each colored line represents the location and length of the OCR respect the closest TSS but the intensity (height) of the peak is not depicted. All four RGC samples came from independent rat litters, two samples per developmental stage. **(B)** Venn diagram representing OCRs that were detected in both E21 samples (116,417), in both P11 samples (131,806), or in all four samples (85,390). **(C)** Pearson correlation coefficient (p < 0.01) between E21 gene RNA expression and the chromatin accessibility value (sum of all OCRs’ normalized peak height values) at the promoter region of genes in E21. **(D)** Pearson correlation coefficient (p < 0.01) between P11 gene RNA expression and chromatin accessibility at the promoter region of genes in P11. **(E)** Pearson correlation coefficient (p < 0.01) between the changes of RNA expression and the changes of chromatin accessibility at the gene promoter regions between E21 and P11. **(F)** Visual representation of chromatin accessibility of E21 and P11 replicates near the TSS of regenerative associated genes *Smad1* (promoter of axon growth) and *Klf9* (inhibitor of axon growth). DORs are represented in blue accordingly to the developmental stage where this region is more accessible.

To determine the changes in chromatin accessibility between embryonic and postnatal RGCs, immunopurified rat E21 and P11 RGCs were processed for ATAC-seq (n = 2 independent rat litters per developmental stage). DNA libraries were sequenced generating 40.9 ± 3.6 million reads. ATAC-seq Integrative Analysis Package (AIAP) was used for sequence quality assurance, mapping, open chromatin region (OCRs) calling, and downstream differential open chromatin analysis^42^. We obtained a total of 33.8 ± 2.5 million mapped reads and 28.5 ± 2.6 million non-redundant unique mapped reads. We identified 150 ± 17.3 thousand OCRs for each sample^43^ and they were enriched near transcription start sites (TSS) (Fig. 2A). PCA analysis of the OCRs present in each of the four samples demonstrated that embryonic and postnatal replicates clustered separately (Supplementary Figure S2A). Pearson correlation coefficient (PCC) between the per-genomic region read count vectors at 1 kb and 5 kb resolution for each pair of samples was used to assess the global similarity between biological replicates. The two biological replicates from each developmental stage were highly correlated at both 1 kb (R > 0.96, P < 2.2e−16, Supplementary Fig. 2B) and 5 kb resolutions (R > 0.97, P < 2.2e−16; data not shown), highlighting the quality and reproducibility of the data. The distribution of all the OCRs in genomic features (promoters, UTR, exon, intron, downstream, and intergenic) were similar among all the samples (Supplementary Fig. 2C).

To ensure robust results in downstream analyses, we only used OCRs that were identified in both biological replicates for each developmental stage. A total of 116,417 OCRs were shared by both E21 samples, and 131,806 OCRs shared by both P11 samples (Fig. 2B). These OCRs, at either E21 or P11, were used to determine whether mRNA expression levels correlate with chromatin accessibility at promoter region (< 3 kb from TSS), genic region (intron and exon), and distal region (> 3 kb but < 100 kb from TSS) at each developmental stage. The value for chromatin accessibility for each gene was defined as the sum of the average normalized peak counts of all the OCRs associated with a gene at each studied region. The chromatin accessibility of the promoter region moderately correlated with the RNA expression at each stage (Fig. 2C,D). Also, the changes in chromatin accessibility at the promoter region between E21 and P11 moderately correlated with the changes in RNA expression between E21 and P11 (Fig. 2E). In contrast, the chromatin accessibility at the genic and distal region correlated very weakly to RNA expression (Supplementary Fig. 2D–I). OCR visualization of selected RAGs, which were differentially expressed at the mRNA level between E21 and P11 (i.e. *Smad1, Myc, Jun, Klf2,* and *Klf9*), confirmed that the promoter region is more accessible in the stage with higher mRNA expression (Fig. 2F and Supplementary Fig. 4).

**Figure 3:**
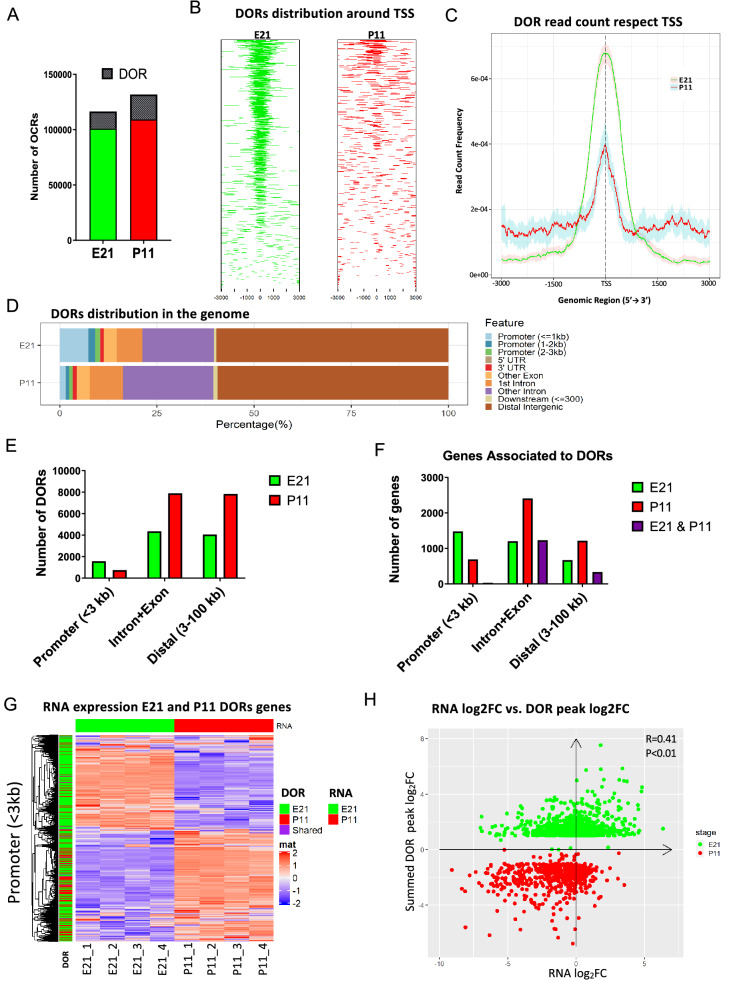
Differentially open regions (DORs) are preferentially located at the promoter region of E21 genes and correlate with RNA gene expression changes. **(A)** The number of OCRs that were present in both samples of each developmental stage and the number of DORs (cross-hatched area) obtained using DESeq2^69^ implemented in the ATAC-seq Integrative Analysis Package (AIAP) package with default parameters. **(B)** Distribution of DORs with respect the closest gene TSS. **(C)** Read count frequency of DORs with respect to TSS. **(D)** Distribution of DORs in the different regions of the genome. **(E)** Number of DORs at the promoter region (< 3 kb from TSS), distal region (> 3 kb but < 100 kb from TSS), and genic (intron + exon) region. **(F)** Genes associated with DORs present at the promoter, distal, and genic region. **(G)** RNA expression changes at E21 and P11 in relation to the presence of DORs at the promoter region. Each row represents a gene that is associated with only E21 DORs (green), only P11 DORs (red), or both E21 and P11 DORs (purple). **(H)** Pearson correlation coefficient (p < 0.01) of log2 fold changes in gene expression between E21 and P11 and log2 fold changes in peak signal of DORs that are located in the promoter regions.

Next, we used DESeq2 implemented in AIAP to determine chromatin regions whose accessibility were statistically different between E21 and P11 (termed differentially opened regions, DORs) (Supplementary Fig. 3A). Of the 116,417 OCRs identified in both of the E21 samples, 15,402 were significantly more accessible at embryonic stage (E21 DORs; Fig. 3A) compared to postnatal. Conversely, of the 131,806 OCRs identified in P11 samples, 22,444 were more accessible at postnatal (P11 DORs; Fig. 3A). Interestingly, E21 DORs were highly enriched at TSS compared to P11 DORs (Fig. 3B,C). More E21 DORs were located within promoters whereas P11 DORs were more prominent in introns compared to E21 (Fig. 3 D). As a result, we identified 1,583 E21 DORs and 747 P11 DORs in the promoter region (< 3 kb from TSS) (Fig. 3E). Genes associated with these DORs were identified, resulting in 1,427 genes associated with E21 DORs (Fig. 3F) including RAGs such as *Smad1, Tubb3, Jun, and Myc* (Fig. 2F and Supplementary Fig. 4). We found 689 genes that were associated with P11 DORs (Fig. 3F) including RAGs such as *Klf9* and *Klf2* (Fig. 2F and Supplementary Fig. 4). Only 29 genes were associated with both E21 and P11 DORs in the promoter region, with chromatin openness changing in opposite directions during development (Fig. 3F). Interestingly, The E21 DOR- associated genes had fewer OCRs at P11 and those OCRs were evenly dispersed across the 3 kb regions surrounding the TSS (Supplementary Fig. 3B). The P11 DOR-associated genes had also fewer OCRs at E21. However, those OCRs were enriched in the TSS regions (Supplementary Fig. 2E). These results demonstrate that the E21 and P11 DOR associated-genes have distinct chromatin accessibility between these two developmental stages.

Next we investigated the relationship between changes of chromatin openness at the DORs and changes in mRNA expression of the DOR-associated genes between E21 and P11. The stage-specific increase of RNA expression levels of the DOR-associated gene is associated with the presence of stage-specific DORs at the promoter region (Fig. 3G) but not DORs at the genic or distal regions (Supplementary Fig. 3C,E). The changes in mRNA expression of the DOR-associated genes and the changes in chromatin accessibility of the DORs have a moderate but significant correlation at the promoter region (Fig. 3G,H). In contrast, we observed a very weak correlation at the genic region and no significant correlation at the distal region (Supplementary Fig. 3C–F).

### TF binding sites (TFBSs) are enriched in developmental stage-specific DORs

**Figure 4:**
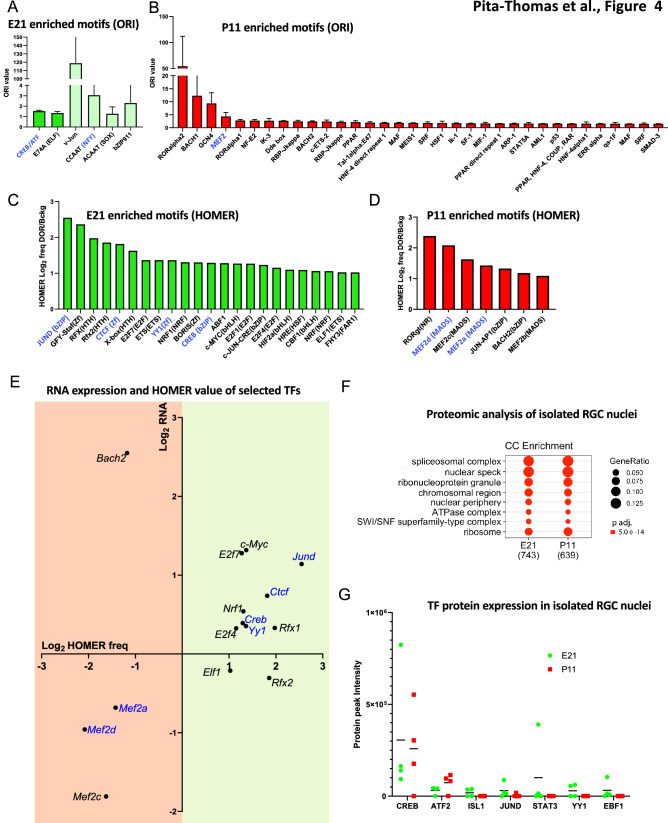
TF Binding site motifs enriched at DORs located in the promoter regions. **(A)** Overrepresentation index (ORI) of TF binding motifs that are exclusively enriched at E21 DORs located in the promoter regions (FDR adjusted p values < 0.01). Deep green indicates binding motifs that appear in greater than 5% of DORs. **(B)** ORI of TF binding motifs that are exclusively enriched at P11 DORs located in the promoter regions. **(C)** TF binding motifs that are exclusively enriched at E21 DORs located in the promoter regions according to the HOMER analysis. **(D)** TF binding motifs that are exclusively enriched at P11 DORs located in the promoter regions according to the HOMER analysis. Only binding motifs that are present in more than 5% of all the DORs in either E21 or P11 and are at least two fold more enriched than the background set are plotted (FDR adjusted p values < 0.01). **(E)** TFs with binding motifs in **(C,D)** that appeared in RNA-seq data were plotted over light green (E21) and light red (P11) background respectively. X-axis: HOMER frequency ratio from **(C,D)**, y-axis: log2 fold change of the transcription factor RNA expression levels between the two stages. HOMER frequency ratio in P11 was represented with a negative value. **(F)** Cellular component GO analysis of proteins identified in RGC nuclei by proteomics. **(G)** Protein peak intensity of selected TFs (from A and B analysis) in E21 and P11 RGC nuclei isolated from rat independent litters (n = 4 for each developmental stage). Protein peak intensity is normalized to total amount of protein of each sample. The black line represents the average of these samples in either E21 or P11 for each TF. Transcription factors in blue in **(A–E)** are those that were detected by proteomic analysis (LC–MS) in RGC nuclei. For ORI of TF binding motifs, the p-value was calculated according to the method described in^75^. Motif enrichment was calculated with cumulative binomial distributions statistics in HOMER^49^.

To identify potential TFs regulating the transcriptional change between E21 and P11, we performed TF binding motif enrichment analysis to identify TFBSs that were enriched in the DORs of either E21 or P11, located in the promoters, since these regions significantly correlated with mRNA expression (Fig. 3G). Overrepresentation index (ORI), which takes into account the frequency and the density of a particular binding motif in a set of target sequences^44^, was calculated to measure how much more probable it is to find a particular TF binding motif in stage specific DORs than in a random background set in the genome. We identified six binding motifs that were exclusively enriched (false discovery rate (FDR) adjusted p values < 0.01) at E21 DORs but not at P11 DORs (Fig. 4A and Supplementary File 3). The TFs associated with these binding motifs include CREB/ATF, E74A (mammalian ortholog: ELF), bZIP911, v-Jun, ACAAT, and CCAAT. However, these last 4 binding motifs appear in less than 5% of the DORs undermining a widespread role regulating the E21 transcriptome. In contrast CREB/ATF and E74A appeared in more than 50% of the DORs supporting an important role in regulating E21 genes (Supplementary File 3). We also found 78 binding motifs that were exclusively enriched in P11 DORs but not in E21 (Supplementary File 3) including MEF2, BACH, and RAR-related orphan receptors (Fig. 4B). Binding motifs of certain TFs were found to be enriched in both E21 and P11 DORs suggesting that they control different genes depending on the cellular context. Some of these binding motifs were more predominant in one particular stage as shown by the ORI ratio (Log2 E21 ORI/P11 ORI; Supplementary Fig. 5 and Supplementary File 3). These TFs include TAX/CREB, NRF-1, E2F, MYC, HIF1, and c-JUN in E21, and LXR in P11. Some of them has been shown to have a role in axon growth such as MYC^45^, HIF1^46^, c-Jun^47^ and NRF-1^48^. This suggests that their activity or the number of genes they regulate may vary during development.

We additionally performed TFBS enrichment analysis using HOMER software^49^ (Supplementary File 4). In this case, the percentage of DORs containing the TF motif is calculated for E21 and P11 DORs and compared to a random background sequence obtained from the complementary regions of the OCRs to calculate statistical differences. Therefore, TF binding motifs that appeared significantly more in DORs than in a background sequence were considered to be enriched in the promoter region (Supplementary File 4). Twenty-three and seven TF motifs for E21 and P11, respectively, were significantly enriched (Benjamini adjusted p values < 0.01) for at least two folds in the DORs compared to the background set (Fig. 4C,D and Supplementary File 4) and appeared in more than 5% of DORs at the specific developmental stage. Interestingly, these TF binding motifs are uniquely enriched in one of the developmental stages which supports their role in controlling programs at a specific developmental stage. We found binding motifs being enriched exclusively in E21 DORs such as CREB, c-MYC, CTCF, YY1, RFX, JUND, NRF1, ELF1, and E2F (Fig. 4C) and other set of binding motifs being enriched exclusively in P11 DORs, such as MEF-2, BACH2, and ROR receptors (Fig. 4D). Other TFs that have been described as pro-regenerative in the literature such as SOX4^8^, HIF1^46^ and STAT3^50^ were also significantly enriched in E21 DORs with frequencies 1.6 to 1.9 times higher than the background set (Supplementary File 4), although is lower than the stringent cutoff we set in Fig. 4C. Interestingly, CREB and MEF2 motifs were found to be uniquely enriched in E21 and P11 respectively, by both methods which utilize different TFBS motif database and very different statistical frameworks. Many TFBS motifs found to be more highly enriched in E21 than P11 by ORI ratio were found to be uniquely enriched in E21 by HOMER, e.g. MYC, JUND, NRF1 and E2F, demonstrating consistent high activity of those TFs in E21. The mRNA expression levels of most TFs correlated with their binding motif enrichment at their given developmental stage (Fig. 4E) supporting the role of TFs, such as CREB, c-MYC, JUND, NRF1, CTCF, E2F7, RFX, YY1 and MEF2 in regulating RGC transcriptional change during development.

### Proteomic analysis of RGC nuclear fractions confirms the presence of TFs regulating transcriptional change

Since the presence of an mRNA does not always guarantee the presence of its corresponding protein, we performed a proteomic analysis of isolated nuclei from E21 and P11 RGCs to identify TFs that are expressed in RGC nuclei at the protein level. A total of six E21 samples and five P11 samples were analyzed by LC–MS (Supplementary File 5). We identified 1192 proteins present in RGC nuclei. GO enrichment analysis showed that these proteins were enriched for nuclear function-related terms (Fig. 4F), confirming that nuclei isolation was efficient, in line with what was previously reported^23^. Uniprot analysis indicated that 72 of the proteins identified have DNA-binding activity and were detected in at least one sample (Supplementary File 5). These proteins included several of the TFs that have enriched binding motifs in DORs such as CREB, JUND, c-MYC, CTCF, YY1, ATF2, MEF2a, and MEF2d (Fig. 4A–D). We next quantified the expression levels of these TFs by normalizing the peak intensity for each TF in LC–MS to the total protein levels in the sample (Fig. 4G).

### CREB binding domain motifs are widespread at the promoter region of E21 DEGs

**Figure 5:**
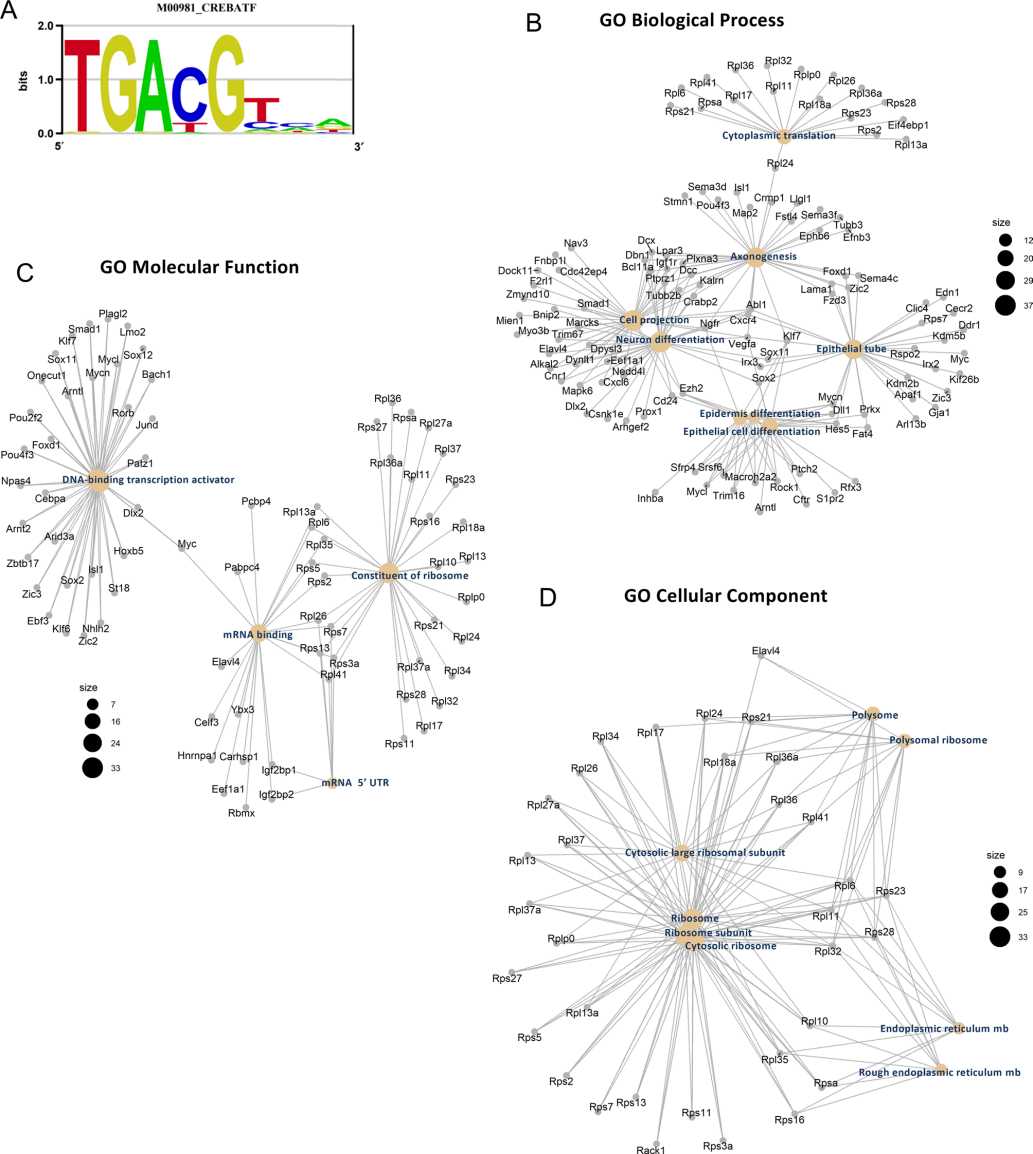
CREB/ATF motif identified at the promoter of genes that are changing during development. **(A)** Sequence motif logo of the CREB/ATF position weight matrix. **(B-D)** Gene hubs obtained by gene ontology analysis of E21 upregulated genes containing CREB/ATF binding motif at their promoter OCRs (For pathways analysis, FDR corrected p value < 0.05 were considered as significant.). **(B)** Eight most significantly enriched biological processes are represented. **(C)** Five most significantly enriched molecular functions are represented **(D)** Eight most significantly enriched cellular components are represented.

Since we detected the presence of CREB and ATF2 at the protein level in RGC nuclei (Fig. 4G) and the CREB/ATF binding motif is enriched at E21 DORs in our binding motif analysis (Fig. 5A), we focused on this binding motif to identify the specific pathways regulated by CREB in E21 RGCs. From the 766 DEGs that have at least one OCR in their promoter, CREB/ATF binding motif appeared in 498 of them. We performed Biological Processes GO analysis for the group of genes with CREB/ATF binding motifs and found that these genes predominantly regulate neuron differentiation, axonogenesis and cytoplasmic translation (Fig. 5B). We next performed Molecular Function GO analysis and found that CREB/ATF binding motif predominantly appears in the promoter of genes that have DNA binding transcription factor activity including regenerative TFs such as SMAD1, KLF7, MYC, or SOX11, and also ribosome genes (Fig. 5C). GO Cellular Component analysis confirmed that many of the genes with CREB/ATF binding motif at their promoter region are ribosomal (Fig. 5D). Altogether, these analyses suggest that CREB occupies a high rank in the hierarchical order of regenerative TFs and also plays an important role regulating ribosome biogenesis.

### CREB-VP64 overexpression promotes optic nerve regeneration

**Figure 6:**
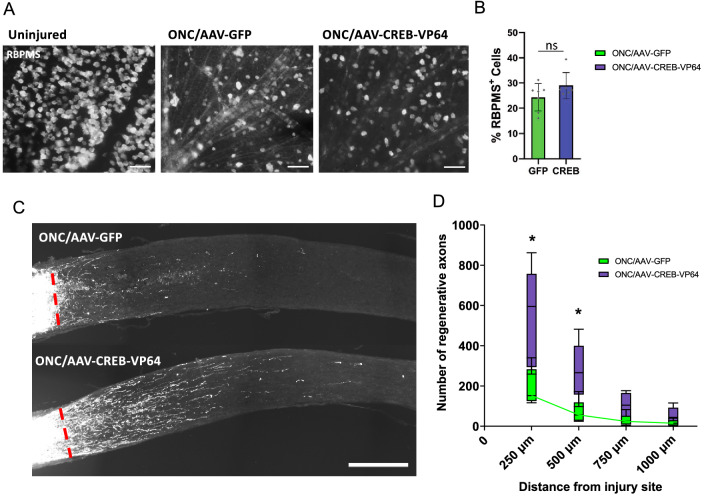
Overexpressing CREB-VP64 promotes optic nerve regeneration. **(A)** Representative images of retinal wholemounts stained for RBPMS (white) to label RGCs in the uninjured and injured condition 2 weeks after optic nerve crush. Scale bars = 50 µm. **(B)** Quantification of RGC survival 2 weeks post crush. Eyes injected with AAV2-CREB-VP64 (n = 7 animals) showed a trend towards increased survival compared to eyes injected with AAV2-GFP that was not significant (unpaired T-test. p = 0.1308). Error bars represent mean ± SEM. **(C)** Representative images of optic nerves sections showing regenerative fibers of mice intravitreally injected with either AAV2-CREB-VP64 or AAV2-GFP. Scale bar = 250 µm. **(D)** Number of regenerative fibers at various distances. Multiple t-test analysis with Holm-Sodak correction. * p adj < 0.05. n = 6 optic nerves per treatment. Four optic nerve sections were used to calculate the average for each nerve. Box-Whiskers plot was used where the box represents the 25th and 75th percentiles, the inside line represents the median, and the whiskers represents the largest and smallest values.

Our ORI and HOMER analysis revealed that CREB binding motifs are enriched in E21 DORs, suggesting that during development, CREB target gene regulation decreases as axon growth capacity declines. Therefore, we tested if overexpressing an active form of CREB would promote axon regeneration. To achieve this, we fused CREB to the VP64 transactivation domain, a tetramer of the 11 amino acids minimal activation domain of VP16, which significantly increases the recruitment of transcriptional machinery and increases activation compared to VP16^51^. Previous studies have demonstrated that the addition of VP16 on pro-regenerative transcription factors increases their ability to drive regeneration *in* *vivo*^9,14,52^. We performed intravitreal injections of AAV-CREB-VP64 fusion, or AAV-GFP as a control, as described previously^23^. This procedure achieves expression of the transgene in ~ 70% of RBPMS positive RGCs (Supplemental Fig. 6). Fourteen days after viral injection, we performed optic nerve crush injury. After another fourteen days, retina were dissected to quantify cell survival or the fluorescently labeled cholera toxin b was injected to trace regenerating axons. We found that overexpression of CREB-VP64 did not significantly increase RGC survival two weeks post optic nerve injury compared to GFP control (Fig. 6A,B). However, overexpression of CREB-VP64 significantly promoted optic nerve regeneration compared to GFP control, tripling the number of regenerating axons (Fig. 6C,D). The extent of axon regeneration we observed two weeks after optic nerve injury is similar to what has been reported by manipulating SOX11, c-Myc or SOCS3^8,27,45^ or by expressing the combination of the transcription factors Oct4-Sox2-Klf4^11^. These results indicate that CREB-VP64 promote RGC axon regeneration, supporting a key role for the CREB-dependent transcription program in the axon growth capacity of RGCs. Further studies will be needed to test if CREB overexpression synergizes with other TFs like ATF2 or other strategies targeting the mTOR signaling pathways^23,29,31,53^ or DNA methylation patterns^11^.

## Discussion

In the present study, we used RNA-seq, ATAC-seq, and proteomics analyses to identify the key TFs regulating RGC axon growth capacity during development. We have shown that the RNA expression of genes that control axon growth and ribosome biogenesis are downregulated during RGC development, and that the mRNA expression changes correlate with chromatin accessibility changes at the promoter region. We found that binding motifs of several TFs that are present at the protein level such as CREB, JUND, CTCF and YY1 are enriched at these chromatin regions. The CREB/ATF motif is especially prevalent, and is enriched at the promoter regions of other regenerative TFs, suggesting a high rank of CREB in the hierarchical order of TFs regulating axon growth. In accordance with this role, overexpressing CREB fused to the VP64 transactivation domain in RGCs promotes optic nerve regeneration to a similar extent as targeting the mTOR pathway^23,29,31,53^, DNA methylation^11^ or expressing the transcription factors Sox11, c-Myc or SOCS3^8,27,45^ or protrudin^26^, partially reverting the poor regenerative capacity of adult RGCs.

Our analysis provides a map of the chromatin accessibility in acutely purified retinal ganglion cells at two different stages of development. Immunopanning enabled us to obtain a sufficient number of cells with purity close to 99% and with good RNA and DNA integrity. A downside is that it takes a few hours to dissociate and isolate the RGCs, which may result in gene expression changes. However, previous studies have shown that those changes are small^54^ and limited to immediate early genes^7^ and thus do not preclude obtaining reliable gene expression data. Since RGCs represent less than 0.5% of all the cells in the retina, our study provides more specific information of the TFs regulating axon growth than studies analyzing whole retina tissue^30,55^. Specifically, we found that the downregulation of genes in postnatal stages, such as axon growth genes and ribosomal genes, correlates with specific promoter regions becoming less accessible. In contrast, genes that regulate synapse function are upregulated during development and their promoter regions become more accessible. Our results are consistent with the observation that in the cortex, there is a developmental change in promoter accessibility that correlates with gene expression^56^. Venkatesh et al. study demonstrates that the target genes of the key RAGs STAT3 and JUN, but not SOX11 and KLF6, become less accessible in the adult stage^56^. This study was however limited by using a heterogenous population of cortical cells, whose proportions are dynamically changing during development. Another advantage of our study is that we complemented our TF binding motif analysis with proteomic analysis of RGC nuclei. This allowed us to confirm the presence at the protein level of several of the TFs whose binding motifs were enriched at DORs such as CREB, JUND, CTCF, YY1, and ATF2. We also observed TFs whose target regions are becoming less accessible during RGC development including TFs with known roles in axon regeneration, such as CREB, JUN, SOX4, c-MYC, CTCF and HIF1^5,8,14,20,45–47^, as well as other TFs whose roles in axon regeneration have not been thoroughly studied such as YY1, NRF1 and RFX. Interestingly, KLF’s motifs were not found to be predominantly enriched at any stage. KLF6 and KLF7 are highly expressed in embryonic stage, whereas KLF2 and KLF9 are highly expressed in postnatal stage^5^, and these TFs have opposing effects in axon growth. Since TFs of the same family bind to similar motifs, it is possible that we were not able to distinguish whether the binding motif present at the DOR is from an axon growth promoting (KLF6 and KLF7) or axon growth inhibitory KLF (KLF9 and KLF2). We also found that the promoter regions of regenerative RAGs such as SMAD1, MYC and TUBB3 were less accessible in P11 RGCs compared to E21. In Zebrafish, a species that can successfully regenerate axons in the optic nerve, chromatin accessibility does not significantly change between non injured RGCs and regenerating RGCs^55^. This suggests that in zebrafish, the chromatin remains fairly open at the target genes of regenerative TFs, whereas in mammals, the chromatin closes during development, limiting the access of regenerative TFs to activate the expression of RAGs after optic nerve injury. Altogether, these results supports the notion that in mammals, axon growth decline might be explained by the limited accessibility of TFs to certain regions of the chromatin.

Our chromatin analysis also showed that the DORs at the promoter of postnatal genes are enriched for certain TF binding motifs, including MEF2. Interestingly, MEF2 isoforms were upregulated in postnatal stage in our RNA-seq data, and MEF2A and MEF2D were also identified at the protein level in postnatal RGC nuclei. This suggests that MEF2 might have an important role in regulating RGC maturation and potentially axon growth inhibition. However, it has been recently shown that deleting MEF2 or overexpressing a MEF2 dominant negative isoform promote RGC survival but not optic nerve regeneration^35^.

It is noteworthy that the expression levels of CREB do not change between E21 and P11 according to our RNA-seq and proteomics data. This contrasts with prior studies showing that CREB is higher in actively growing RGCs compared to those that have already reached their targets^6^. Our approach does not allow us to differentiate between those two developmental states, which may explain why the higher expression at E21 in our data is unable to reach statistical significance. Another possibility is that differences in axon growth capacity between the two stages may be not entirely relate to a higher expression of CREB but also to a higher activation state of CREB in RGCs. Our chromatin accessibility analysis allowed us to identify CREB motifs in DORs suggesting that this TF is regulating transcriptional changes in embryonic stage. CREB activity is regulated by cAMP levels and previous studies have shown that the levels of cAMP drop after birth during CNS and PNS neuron development^57^. In rat RGC, cAMP levels are three times higher in E18 compared to P5^57^. This suggests that CREB is activated and regulating axon growth genes in embryonic RGCs. After birth, cAMP levels drop and therefore transcription of axon growth genes by CREB ceases. Interestingly, activating CREB by increasing cAMP levels *in vivo* has shown controversial results. Some studies reported modest increase in optic nerve regeneration^58^, whereas others showed that increasing cAMP alone was not sufficient to promote optic nerve regeneration, but could when combined with other stimuli such as providing neurotrophic factors^59^, oncomodulin or PTEN deletion^13^. In addition, elevating cAMP in cultured RGCs by adding forskolin does not fully revert the axon growth decline observed in postnatal RGC^4^. We observed that the chromatin in the promoter region of axon growth promoting genes is less accessible in postnatal RGCs, suggesting that endogenous CREB activated by cAMP may be unable to effectively access these chromatin sites to promote regeneration in adult RGCs^15^. We demonstrate that expression of CREB fused to the transactivation domain VP64 in adult RGCs promotes optic nerve regeneration. Therefore, CREB-VP64, but not endogenous CREB activated by cAMP, might be able to open the chromatin of pro-regenerative CREB target genes to induce optic nerve regeneration. Transactivation domains such as VP16 and VP64 are well-known chromatin-modifying agents that open chromatin and greatly activate transcription^60^. However, it is possible that other TFs in combination with CREB-VP64 could further increase the number of regenerating RGC and the distance at which they regenerate. Combining CREB-VP64 with other TFs or treatments targeting the neurotrophic factor CNTF^61,62^, mTOR^23,29,31,53^ or DNA methylation^11^ might provide a synergistic effect in optic nerve regeneration.

## Methods

### Retinal Ganglion cell immunopanning purification

All animal protocols were approved by the Washington University School of Medicine Institutional Animal Care and Use Committee (IACUC) under protocol A3381-01. All experiments were performed in accordance with the relevant guidelines and regulations. All experimental protocols involving rats and mice were approved by Washington University School of Medicine (protocol #20180128). Mice and rats were housed and cared for in the Washington University School of Medicine animal care facility. This facility is accredited by the Association for Assessment & Accreditation of Laboratory Animal Care (AALAC) and conforms to the PHS guidelines for Animal Care. Accreditation—7/18/97, USDA Accreditation: Registration # 43-R-008. The study was carried out in compliance with the ARRIVE guidelines^63^.

The isolation of RGCs was performed by using a slightly modified version of the immunopanning procedure originally established in Barres laboratory^64^. For embryonic RGCs, E21 day pregnant Sprague Dawley rats were euthanized by CO_2_ and embryos were transferred to a plate with DPBS where retinas were dissected out and cleaned. For postnatal RGCs, P11 Sprague Dawley pups were euthanized by CO_2_ and eyes were transferred to DPBS medium where retinas were dissected. Next, RGCs were isolated as previously described^23^. Briefly, retinas were transferred to a filtered solution of papain in DPBS containing 2 mg of L-Cysteine and 2000 units of DNAse. For E21 retina, 140 units of papain were added. For P11, 200 units were added. After 30 min, enzymatic solution was removed and retinas were triturated to obtain a single cell solution. RGCs were isolated by immunopanning. First by negative selection using an anti-macrophage antibody and then positive selection using anti-thy1 antibody. RGCs attached to the plate were trypsinized, counted in a Neubauer, and centrifuged for subsequent processing. The procedure of dissociating and panning cells, which lasts 3–4 h, may result in gene expression changes. However, previous studies have shown that gene expression changes during tissue dissociation are small^54^ and limited to immediate early genes^7^. Therefore this technique is suitable for comparing the transcriptome of the purified cells in two different developmental stages.

### RNA sequencing and analysis

Total RNA from 700,000 to 1.25 million RGCs from four independent rat litters per developmental stage was isolated using the Qiagen RNAeasy kit from Qiagen, which included a 15 min On-column DNAse step. RNA was stored at − 80 °C and sent to Genome Technology Access Center at Washington University for library preparation and sequencing. RNA quality was assessed using an Agilent Bioanalyzer (RIN > 9.5). Samples were subjected to DNase treatment. rRNA depletion was achieved with the Ribo-Zero rRNA removal kit. Library preparation was performed using the SMARTer kit (Clontech), and sequencing performed on an Illumina HiSeq3000. Basecalls and demultiplexing were performed with Illumina’s bcl2fastq software and a custom python demultiplexing program with a maximum of one mismatch in the indexing read. Sequences were adapter-trimmed using Cutadapt 1.16^65^ and subjected to quality control using PRINSEQ 0.20.4^66^ and aligned to Rat (Rattus norvegicus) annotations based on genome assembly RNOR6 using STAR 2.5.3a^67^. Reads in features were counted using HTseq 0.6.1^68^. Genes differentially expressed between conditions were identified using DESeq2^69^ with log2FC > 1.0 and a false discovery rate (FDR) adjusted p values < 0.01, which includes a Benjamini–Hochberg correction^69^. Variance stabilizing transformation (VST) normalized counts were calculated using DESeq2, and normalized gene counts were converted to Z scores for plotting. Heatmaps were generated using ComplexHeatmap R package^70^. Sequencing performance was assessed for total number of aligned reads, total number of uniquely aligned reads, genes and transcripts detected, ribosomal fraction, known junction saturation, and reads distribution over known gene models with RSeQC 2.6.24^71^. R package clusterProfiler^72^ was used for GO and KEGG pathway enrichment analysis and plotting. GO and KEGG pathway terms with FDR corrected p value < 0.05 were considered as significant. R version 4.0.4 was used for statistical analysis and plotting.

R (citation: R Core Team (2013). R: A language and environment for statistical computing. R Foundation for Statistical Computing, Vienna, Austria. http://www.R-project.org/).

### ATAC-Seq and data analysis

After isolation, 300,000 RGCs from two independent rat litters per developmental stage were centrifuged and resuspended in Neurobasal/B27 + medium and 10% DMSO, to quick frozen at − 80 °C. Samples were processed by UCSD Center of Epigenomic Technologies using their proprietary assay for Transposase-Accessible Chromatin coupled with high-throughput sequencing (ATAC-seq). ATAC-seq Integrative Analysis Package (AIAP) was used for sequence quality assurance, mapping, open chromatin region (OCR) calling^42^. We used DESeq2^69^ implemented in the ATAC-seq Integrative Analysis Package for downstream differential open chromatin region identification. Those OCRs that have FDR adjusted p value < 0.01 and log twofold change > 1 were considered DORs (differentially open regions). Following data quality control analyses were performed for each sample and across the projects: (1) Reads under peak percentage ranged 31.6–39.3%. (2) Signal enrichments around the TSS relative to genome wide average, a metric which identifies datasets with high signal to noise ratios, ranged 10.8–14.4%. (3) Pearson correlation coefficient between the two replicates were calculated to measure the concordance between the two biological replicates. To ensure the robustness of our analysis we used only OCRs that were present in both biological replicates of either stage for downstream analyses. This ensures that only the most robust peaks were included in the analyses.

### Transcription factor binding motif enrichment analysis and CREB target analysis

To identify candidate transcription factors that regulate differentially expressed genes, the HOMER^49^ and motif over-representation index (Motif-ORI)^44^ algorithms were used to identify transcription factor binding sites enriched in the differentially open region. Genomic regions complementary to the region of all the called OCRs (considered as all of the non-open regions in the genome) were obtained and were provided as the background set sequence for HOMER analysis. This non-open region set was used as input to perform random sampling of genomic regions to obtain a set of genomic regions with the same number and length distribution as a set of query sequences. This random sampled genomic region sequences were provided as the background set sequence for Motif-ORI analysis and this analysis is repeated 100 times and statistical significance was calculated using a Student's t-test with the NULL hypothesis that ORI is not significantly larger than 1.2, a stringent cutoff determined in previous publication^44^ to distinguish enriched motifs. A motif with Benjamini–Hochberg procedure corrected p value < 0.05 were considered as significant. The TF logo image was generated using enoLOGOS^73^.

To identify targets of CREB, sequence of OCR peaks were retrieved using BEDTools^74^ and was scanned using Patser to identify CREB binding sites. The Patser program calculates the probability of observing a sequence with a particular score or greater^75,76^ for the given matrix and determines the default cutoff score based on that p-value. Any gene with at least one CREB binding site in one of the gene-associated OCR peaks was counted as target gene.

### RGC nuclei proteome characterization by LC–MS

Nuclei from 700,000–1.25 million RGCs from 6 E21 and 5 P11 independent rat litters were isolated using the Thermo Scientific NE-PER Nuclear and Cytoplasmic Extraction Kit. Next, trypsin digestion was perform by adding a buffer containing 100 mM ammonium bicarbonate, 10 mM TCEP and 25 mM iodoacetamide followed by digestion with trypsin at 37 °C overnight. The digested sample was acidified with 1%TFA then cleaned up with C18 tip. The extracted peptides were dried down. Proteomic samples were fractionated in a StageTip casted with four SDB-RPS disks (3 M Empore SPE disks). Peptides were sequentially eluted with four buffers of increasing salt content. The four fractions were dried under vacuum and dissolved with 0.1% formic acid for LC–MS analysis. Fractions of each sample were analyzed by LC–MS with a Dionex RSLCnano HPLC coupled to an Orbitrap Fusion Lumos (Thermo Scientific) mass spectrometer using a 2 h gradient. Peptides were resolved using 75 µm × 50 cm PepMap C18 column (Thermo Scientific). Sequence mapping and label-free quantification were achieved using MaxQuant (version 1.6.1). MaxQuant was set up to search Human reference proteome (Uniprot.org). The digestion enzyme was set as trypsin. Carbamidomethylation of cysteine was set as fixed modification. Oxidation of methionine and acetylation of N-terminal of protein were specified as variable modifications.

### Optic nerve regeneration assay

All surgical procedures were performed under isofluorane anesthesia according to approved guidelines by the Washington University in St. Louis School of Medicine Institutional Animal Care and Use Committee. To generate AAV constructs, GFP or CREB (human sequence) were subcloned into an AAV transfer plasmid provided by the Washington University Viral Core which uses the CMV promoter. AAV2 viral particles were generated by Washington University Viral Core with titers for AAV2-CREB-VP64 (2.4 × 10^12^ vg/ml) and AAV2-GFP (1.7 × 10^13^ vg/ml). Five week old C57Bl/6 mice were anesthetized with isoflurane and intravitreally injected with 1.25 µl of the AAV2-CREB-VP64 or AAV2-GFP virus two weeks before optic nerve crush using a Hamilton syringe and a pulled glass attached by an adaptor. Optic nerve crush was performed by a surgeon blinded to treatment as previously described^23^. Mice were anesthetized by isoflurane inhalation, and optic nerve was exposed and crushed for 3 s with a 55 forceps. For analgesia, 1 mg/kg buprenorphine SR-LAB (ZooPharm) was administered subcutaneously. Two weeks after surgery, mice were intravitreally injected with 1.5 µl of 1 mg/ml fluorescent Cholera toxin B. Two days after, animals were sacrificed by CO_2_ inhalation and perfused with PBS followed by 4% Paraformaldehyde. Nerves were dissected out and post-fixed for 4 h, washed in PBS and incubated in 30% Sucrose solution overnight at 4 Celsius. Nerves were then dissected and sectioned at 11 µm thickness. Optic nerve sections were imaged in a florescent microscope. The number of axons growing at 250, 500, 750 and 1000 µm distal from the injury site were quantified by a researcher blinded to treatment. Multiple t-test analysis with Holm-Sidak correction was performed.

For RGC survival assay, eyes were dissected out of the orbit with Vanna scissors and immersed in 4% PFA overnight at 4 °C. Eyes were transferred to PBS and the cornea, iris, lens, sclera, choroid were removed and the whole retina collected in a 24-well plate under a dissection microscope as described^77^. Following five 5-min washes with PBS, TBST blocking buffer (10% normal donkey serum, 0.5% TritonX-100 in PBS) was applied for 3 h at room temperature with gentle rotation. This was replaced with anti-RBPMS primary antibody (generous gift by Dr. Philip Williams, Cat# ABN1376, EMD Millipore, USA; 1:500) or anti-GFP in TBST blocking buffer at 4 °C overnight with gentle rotation. Following five further 5-min washes with PBS, retina were incubated with Alexa Fluor 594 secondary antibody (1:300) in TBST blocking buffer at room temperature for 2.5 h with gentle rotation. After five further washes with PBS, retina was flattened with four incision and mounted onto charged Superfrost microslides using ProLong Gold antifade mounting reagent (Invitrogen) and allowed to dry overnight at 4 °C. Images of flat mount retina (5 images/flat mount, 1–2 images/quadrant) were acquired using epifluorescence microscope Nikon Eclipse Ti2 with a 40 × objective. Blinded manual counting of all images was performed (100µm^2^ area in each count and 5 count/image). Uninjured eyes were counted (*n* = 3–4 per group) and the averaged used to normalize the percentage RGC survival.

To count GFP positive RGC, three images of each flat mount retina were used to count GFP positive and RBPMS positive cells and the % of GFP positive cells was calculated with respect to total RBPMS positive cells in each image.

## Supplementary Information

Supplementary Figures.

Supplementary Dataset 1.

Supplementary Dataset 2.

Supplementary Dataset 3.

Supplementary Dataset 4.

Supplementary Dataset 5.

## Data Availability

The raw FASTQ files were deposited at the NCBI GEO database under the accession number GSE163564.
