# Supplementary material for: Genome-wide chromatin accessibility analyses provide a map for enhancing optic nerve regeneration
Source: Sci Rep. 2021 Jul 21;11:14924. doi: 10.1038/s41598-021-94341-y (PMC8295311; doi:10.1038/s41598-021-94341-y)
Supplement: Supplementary file 1 — Supplementary Figures. [file 41598_2021_94341_MOESM1_ESM.pdf]

SUPPLEMENTARY INFORMATION

Genome-wide chromatin accessibility analyses provide a map for enhancing optic nerve regeneration

Wolfgang Pita-Thomas[1], Tassia Mangetti Gonçalves[1], Ajeet Kumar[1], Guoyan Zhao[1,2*] and Valeria Cavalli[1,3,4*]

1. Department of Neuroscience, Washington University School of Medicine, St Louis 63110, Missouri, USA
2. Department of Pathology and Immunology, Washington University School of Medicine, St Louis 63110, Missouri, USA
3. Hope Center for Neurological Disorders, Washington University School of Medicine, St. Louis, Missouri 63110, USA
4. Center of Regenerative Medicine, Washington University School of Medicine, St. Louis, Missouri 63110, USA

*co-correspondence to:

Valeria Cavalli, Department of Neuroscience, Washington University School of Medicine, Campus Box 8108, 660 S. Euclid Ave, St. Louis, MO 63110-1093 Phone: 314 362 3540, Fax: 314 362 3446, E-mail: cavalli@wustl.edu

Guoyan Zhao, Department of Neuroscience, Washington University School of Medicine, Campus Box 8108, 660 S. Euclid Ave, St. Louis, MO 63110-1093 Phone: 314 362 9045, Fax: 314 362 3446, E-mail: gzhao@wustl.edu

Running Title: A road map for axon regeneration in RGC

SUPPLEMENTARY FIGURES

Supplementary Figure 1. RNA-seq data of E21 and P11 RGCs. A) PCA analysis of E21 and P11 RNA-seq samples. B) Volcano plot representing fold change between E21 and P11 and p-value. Cut off is Log2 FC>1 and false discovery rate (FDR) adjusted p<0.01. Blue, genes downregulated in P11, and, red, genes upregulated in P11. C) The average (± SEM) RNA expression of pan RGC and RGC subtype markers, and other retinal cell markers in E21 and P11 RGC RNA samples (n=4 RGC preparations from independent rat litters for each developmental stage).

Supplementary Figure 2. ATAC-seq analysis of E21 and P11 RGCs. A) PCA analysis of open chromatin regions (OCRs) of E21 and P11 samples. B) Pearson correlation coefficient analysis of chromatin accessibility signal between the two biological replicate samples from the same stage at 1kb resolution. C) Proportion of OCRs located within the different genomic features in E21 and P11 RGCs. D) Pearson correlation coefficient between E21 RNA expression and chromatin accessibility at the genic region (intron + exon) of genes in E21. E) Pearson correlation coefficient between P11 gene RNA expression and chromatin accessibility at the genic region (intron + exon) of genes in P11. F) Pearson correlation coefficient between the change in RNA expression between E21 and P11 and the change in chromatin accessibility at the genic region (intron + exon) of genes between those stages. G) Pearson correlation coefficient between E21 RNA expression and chromatin accessibility at the distal region (>3 kb but <100Kb) of genes in E21. H) Pearson correlation coefficient between P11 gene RNA expression and

chromatin accessibility at the distal region of genes in P11. I) Pearson correlation coefficient between the change in RNA expression between E21 and P11 and the change in chromatin accessibility at the distal region of genes between those stages.

Supplementary Figure 3. Differentially open chromatin regions and its correlation with RNA expression in RGCs. A) Volcano plot representing the fold change of OCRs between E21 and P11 and the log10 of FDR adjusted p value. OCRs with FDR adjusted p value < 0.01 and log 2 fold change > 1 as determined by DESeq2 implemented in AIAP software package were considered as DORs. E21 DORs represented in red while P11 in blue B) The P11 OCRs from the E21 DOR associated genes of Figure 3B and Figure 3F are shown in the left while the E21 OCRs of the P11 DOR associated genes are shown in the right.C). RNA expression changes at E21 and P11 in relation to the presence of DORs at the genic region. Each row represents a gene that is associated with only E21 DORs (green), only P11 DORs (red), or both E21 and P11 DORs (purple). D) Pearson correlation coefficient of peak signal changes in DORs that are located in the genic region and changes in gene expression of DOR-associated genes between E21 and P11 and. E). RNA expression changes at E21 and P11 in relation to the presence of DORs at the distal region. Each row represents a gene that is associated with only E21 DORs (green), only P11 DORs (red), or both E21 and P11 DORs (purple). F) Pearson correlation coefficient of peak signal changes in DORs that are located in the distal region and changes in DOR-associated gene expression between E21 and P11.

Supplementary Figure 4. Visual representation of chromatin accessibility of E21 and P11 replicates near the TSS of regenerative associated genes *GAP43, Myc, Tubb3, Jun, Klf6, Klf7,* and *Sox11* (promoters of axon growth) and *Klf2* (inhibitor of axon growth). DORs are represented in blue accordingly to the developmental stage where this region is more accessible.

Supplementary Figure 5. Comparison of TF Binding Motifs that were enriched at both E21 and P11 DORs, and were located within the promoter regions. A) Bar plot of log2 of the ratio of the E21 ORI /P11 ORI which represents the relative enrichment of a TF binding motif in E21 compared to P11. Only TF binding motifs with a $\log_2$ (E21 ORI/P11 ORI)>1 or <-1 are plotted. Some TFs have multiple position weight matrices enriched and the one with highest absolute ORI ratio value was plotted.

Supplementary Figure 6. Transduction efficiency of AAV-GFP. A) Representative images of retinal whole mounts from injured eyes previously injected with AAV2-GFP that were stained for RBPMS (white) and anti-GFP. Scale bars = 100 µm. B) Percentage of double positive RGCs (RBPMS+. GFP+) compared to the total number of RGCs (RBPMS+). Error bars represent mean ± SEM (n=5)

SUPPLEMENTARY DATA FILES

Supplementary File 1. RNAseq analysis

Supplementary File 2. GO and KEGG analysis of RNA expression

Supplementary File 3. Promoter 3kb ORI analysis

Supplementary File 4. Promoter 3kb Homer analysis

Supplementary File 5. Proteomics analysis

Pita-Thomas et al., Figure S1

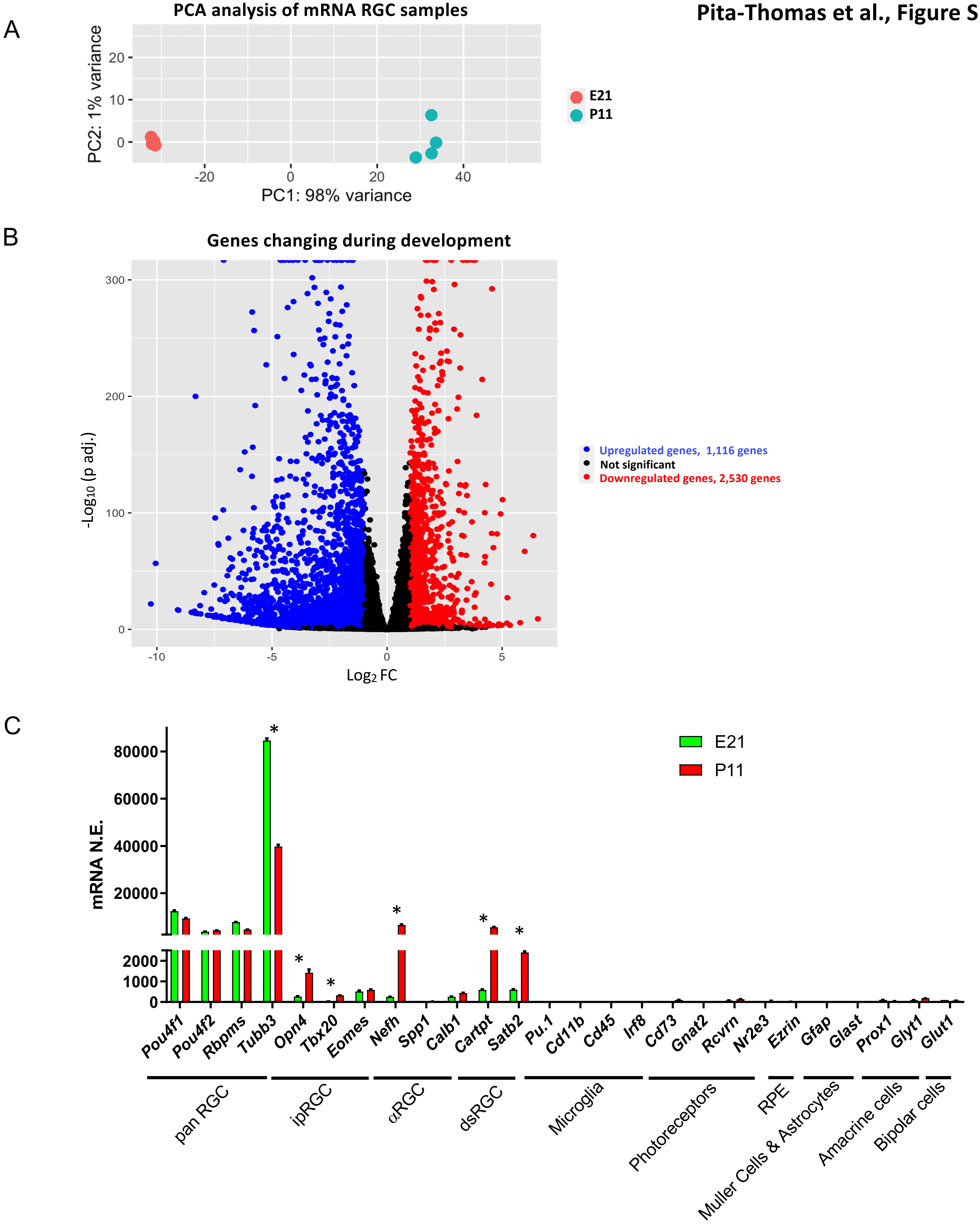

Pita-Thomas et al., Supplementary Fig S2

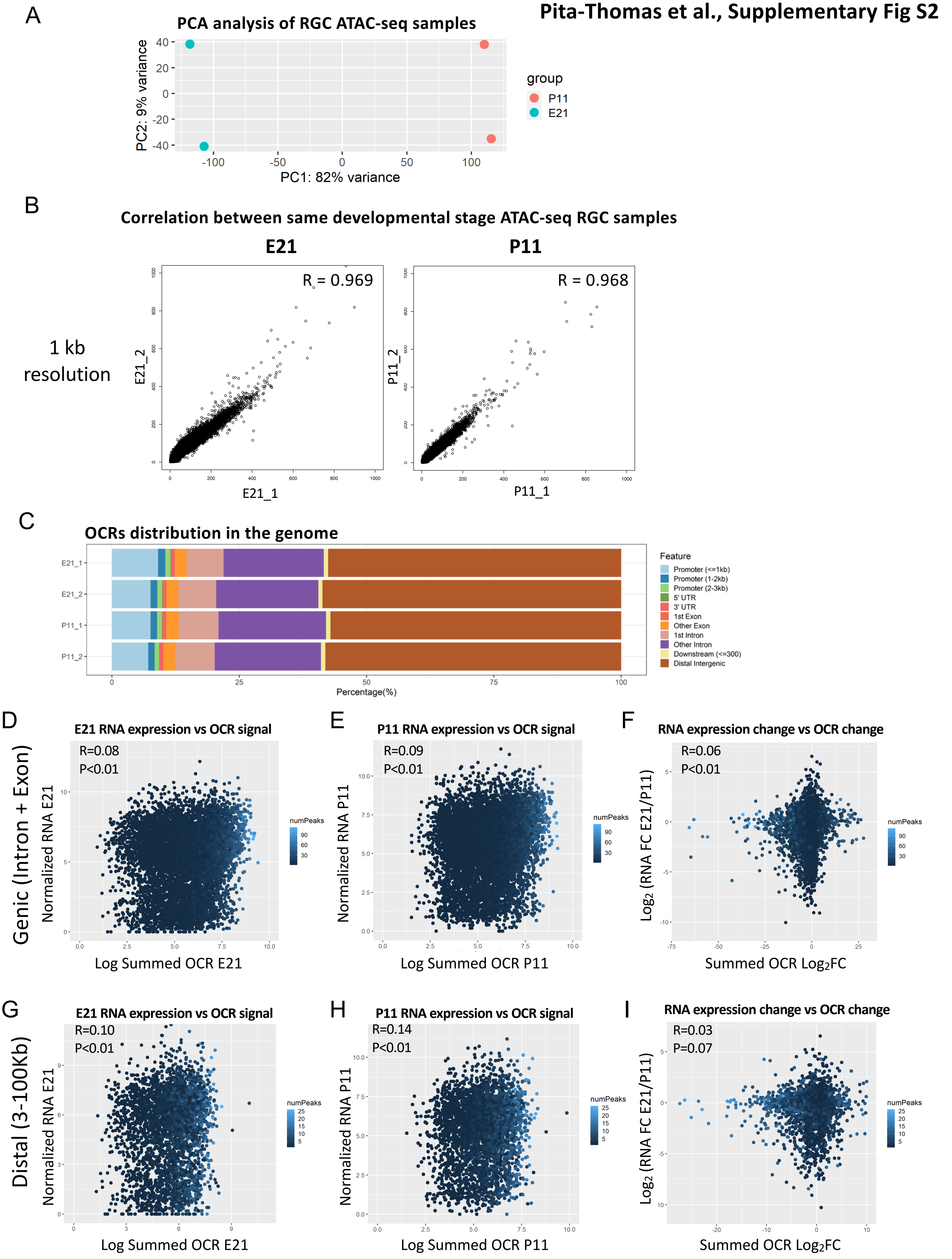

Pita-Thomas et al., Supplementary Fig S3

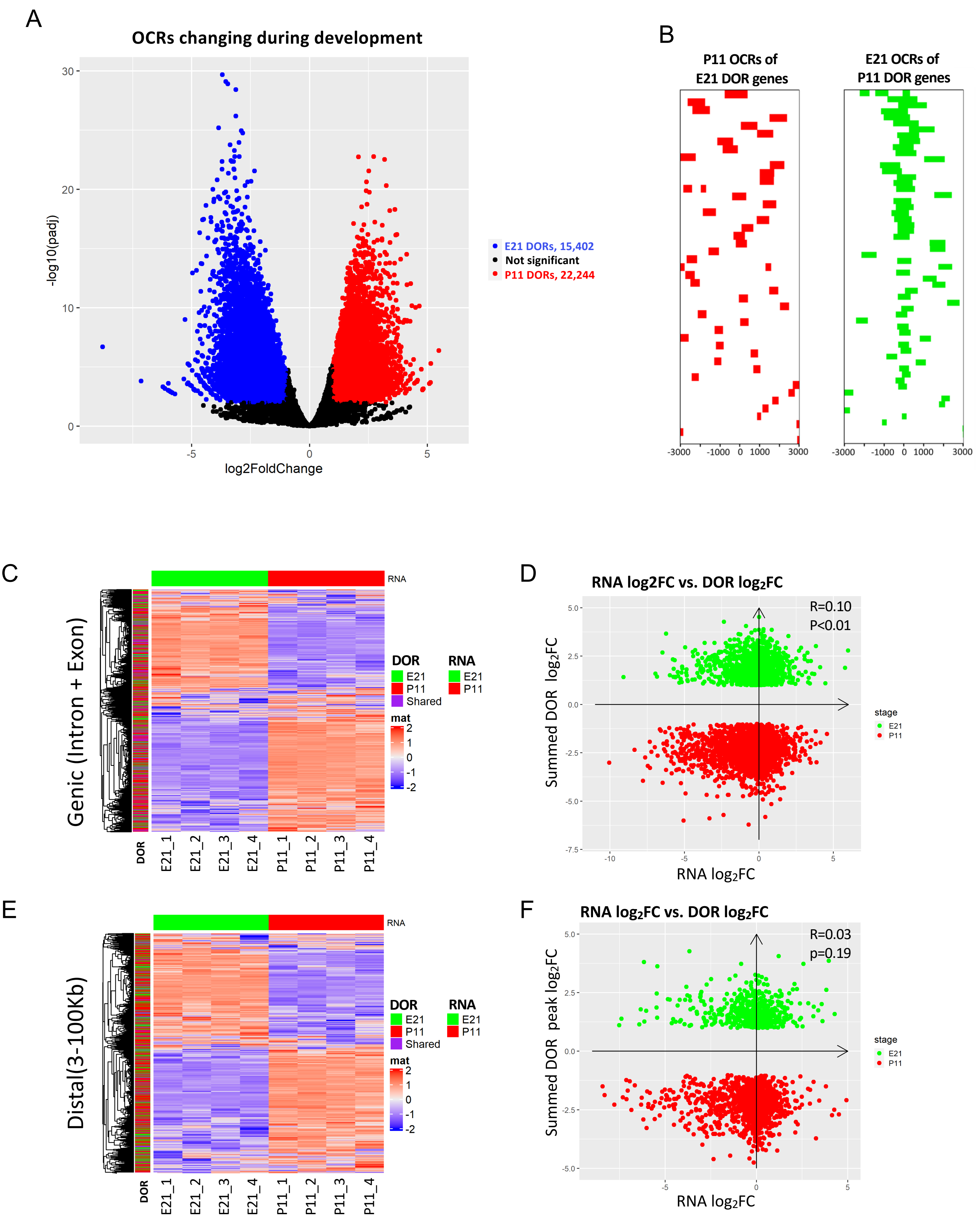

Pita-Thomas et al., Supplementary Fig S4

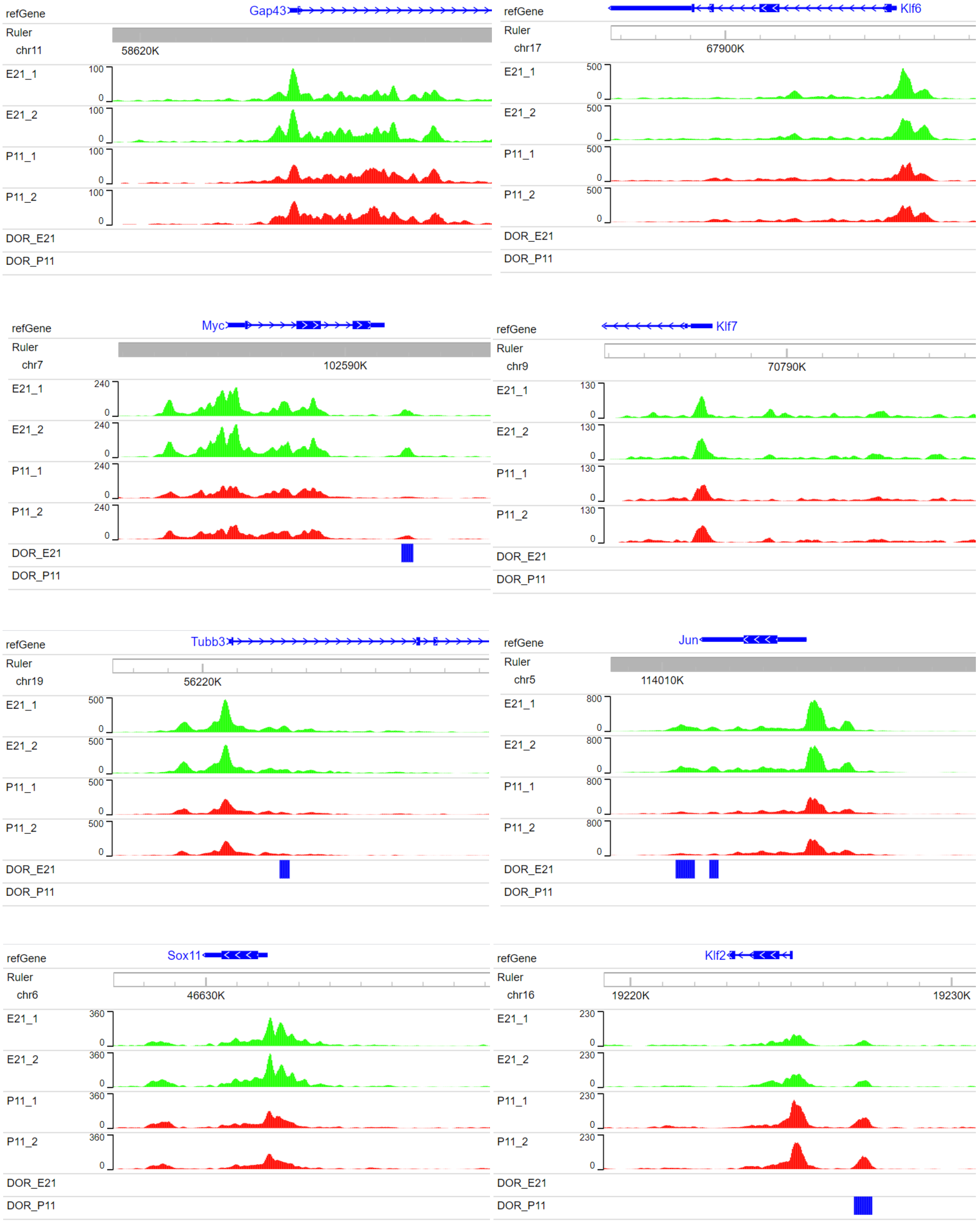

Pita-Thomas et al., Supplementary Fig S5

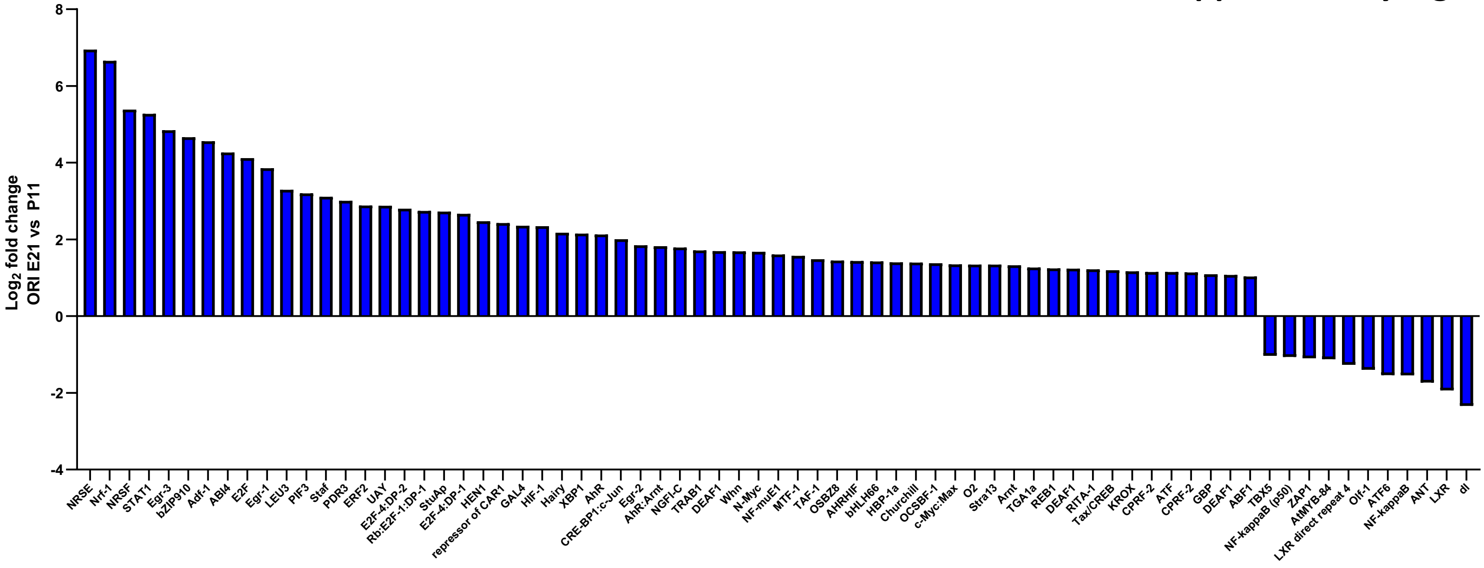

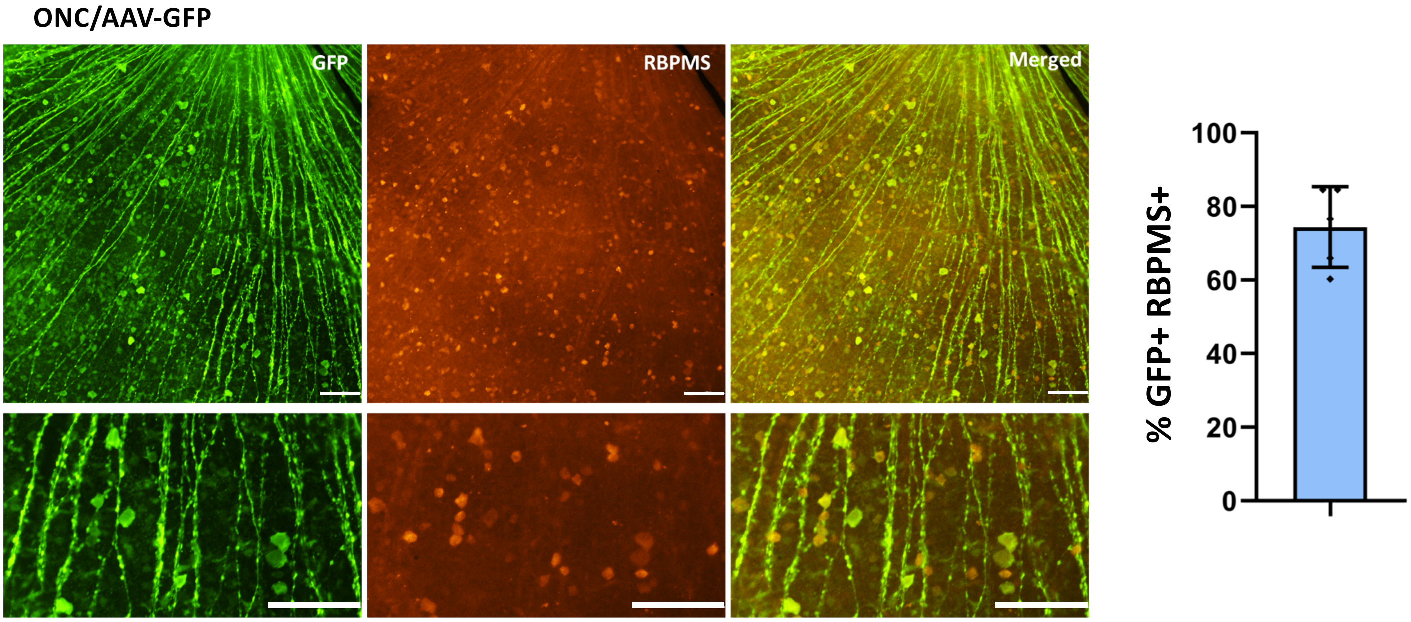
ONC/AAV-GFP
GFP
RBPMS
Merged
% GFP+ RBPMS+
100
80
60
40
20
0
